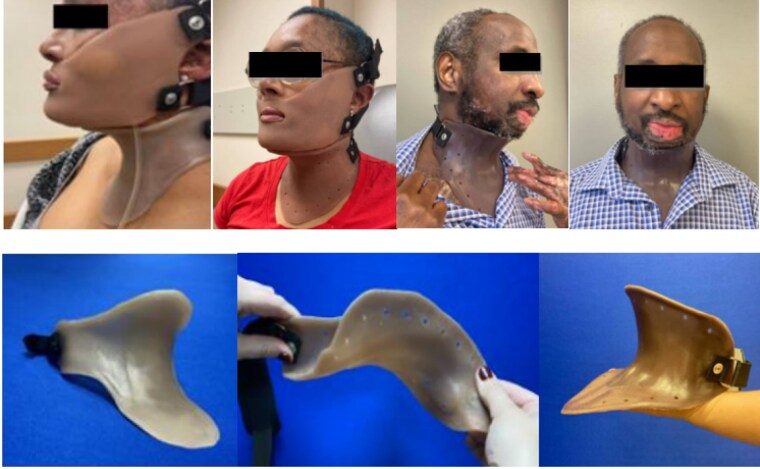# 695 Impact of Semirigid Silicone Neck Collars in Burn Patients over One Year

**DOI:** 10.1093/jbcr/iraf019.324

**Published:** 2025-04-01

**Authors:** Michelle Dwertman, Dr Henry Huson

**Affiliations:** University of Cincinnati Medical Center Burn Center; University of Cincinnati Medical Center Burn Center

## Abstract

**Introduction:**

The management of neck motion and scarring, particularly in burns, is a critical challenge due to the limitations of traditional rigid neck orthoses, which lack the ability to adapt to neck and jaw movements, unable to combine with rigid facemasks, and are hard to customize—leading to poor patient adherence. To address these obstacles, we developed a ‘Sequential Compression Appliance to Reduce Scarring’ (SCARS) neck collar, a novel orthosis designed to provide effective scar management while allowing natural neck movement, and the ability to be combined with a rigid facemask. This study builds on our previous work by further evaluating the SCARS semirigid neck collar and its clinical outcomes in neck range of motion (ROM) and scarring over a one-year period.

**Methods:**

Two burn patients, status post neck grafting, were identified for the study and fitted with SCARS semirigid neck collars. The SCARS neck collar, fabricated with high-consistency rubber (HCR) silicone, is pliable and provides at least 20 mmHg of pressure while allowing full neck and jaw movement. Additionally, the collar is color-matched to the patient’s skin tone for enhanced aesthetic integration and adherence. Key outcomes measured over one year included neck ROM and scar assessments and comfort using the Vancouver Scar Scale and Likert Scale.

**Results:**

While wearing the SCARS collars, patients experienced significant improvements in neck ROM, with an average increase of 25%, and a notable reduction in scarring, including a 15% decrease in scar height and an 12% improvement in pliability. Likert scale scores indicated a strong preference for the semirigid collars, particularly during both eating and sleeping. Patients sleeping with the SCARS collar reported mild difficulty (average: 2.67). Most notably, eating was impossible with the rigid collar (average: 5.00), but the SCARS collar allowed for mild difficulty (average: 2.33).

**Conclusions:**

The SCARS semirigid silicone neck collar is a safe and effective alternative to traditional rigid orthoses for burn patients with scarring. The collar provides essential pressure for scar management while allowing greater neck mobility, leading to possible improved patient adherence and comfort.

**Applicability of Research to Practice:**

The SCRAS neck collar provides a promising alternative to traditional rigid orthoses, offering improved neck mobility while effectively managing scarring in patients. This design allows for greater patient comfort and cosmesis, is able to be worn with rigid facemasks, offers flexibility of movement without compromising the therapeutic goals of managing hypertrophic scarring. The ease of modification with the SCRAS by scissors allows improved access for both patients and providers. Future research should explore the long-term benefits and potential applications of this innovative design in various rehabilitation contexts.

**Funding for the Study:**

N/A